# Early events in speciation: Cryptic species of *Drosophila aldrichi*

**DOI:** 10.1002/ece3.2843

**Published:** 2017-05-03

**Authors:** Cynthia Castro Vargas, Maxi Polihronakis Richmond, Mariana Ramirez Loustalot Laclette, Therese Ann Markow

**Affiliations:** ^1^ Laboratorio Nacional de la Genomica de BiodiversidadCINVESTAVIrapuatoGuanajuatoMexico; ^2^ Division of Biological SciencesUniversity of California at San DiegoLa JollaCAUSA

**Keywords:** cryptic species, *Drosophila aldrichi*, *Drosophila wheeleri*, Mexico, phylogenetic relationships

## Abstract

Understanding the earliest events in speciation remains a major challenge in evolutionary biology. Thus identifying species whose populations are beginning to diverge can provide useful systems to study the process of speciation. *Drosophila aldrichi*, a cactophilic fruit fly species with a broad distribution in North America, has long been assumed to be a single species owing to its morphological uniformity. While previous reports either of genetic divergence or reproductive isolation among different *D. aldrichi* strains have hinted at the existence of cryptic species, the evolutionary relationships of this species across its range have not been thoroughly investigated. Here we show that *D. aldrichi* actually is paraphyletic with respect to its closest relative, *Drosophila wheeleri*, and that divergent *D. aldrichi* lineages show complete hybrid male sterility when crossed. Our data support the interpretation that there are at least two species of *D. aldrichi,* making these flies particularly attractive for studies of speciation in an ecological and geographical context.

## 1 Introduction

Understanding the earliest events in speciation remains a challenging problem in evolutionary biology. According to the Biological Species Concept, species are groups of actually or potentially interbreeding natural populations, which are reproductively isolated from other such groups (Dobzhansky, 1937; Mayr, 1942). The development of reproductive isolation thus is the main feature in the speciation process in eukaryotes. Reproductive isolating mechanisms fall into three categories: (1) premating (sexual), (2) postmating–prezygotic or gametic, and (3) postzygotic (Coyne & Orr, 2004; Dobzhansky, 1937). Several unresolved questions remain regarding the formation of new species that can only be resolved by examining a large number of species in the early stages of speciation. One question is whether one of these isolating mechanisms tends to arise before the others. Another is whether a particular degree of genetic differentiation is observed before the isolating mechanism can be detected.

Flies of the genus *Drosophila* have provided popular model systems to study speciation. Coyne & Orr (1989a, 1989b, 1997) attempted to examine the above questions in meta‐analyses of allozyme data and reciprocal crosses among species. While informative, the taxa they utilized already were recognized as different species and the allozymes have since been replaced with more modern molecular approaches. Well‐established phylogenetic relationships of hundreds of species, for which we also know the resource ecology and geographic distributions, allow us to study speciation at earlier temporal scales. In some cases, the phylogenetic and ecological data direct us to species that are in the early stages of speciation so that we can address the above two questions. The repleta species group in the subgenus Drosophila provides an interesting example of species having recently diverged or still diverging, often in association with shifts in the species of cacti, which, when necrotic, serve as their host plants. One repleta group species, *D. mojavensis*, exists as four different subspecies (Pfeiler, Castrezana, Reed, & Markow, 2009), some of which show early signs of premating (Zouros & D'Entremont, 1980) and postmating–prezygotic isolation (Knowles & Markow, 2001).

**Figure 1:**
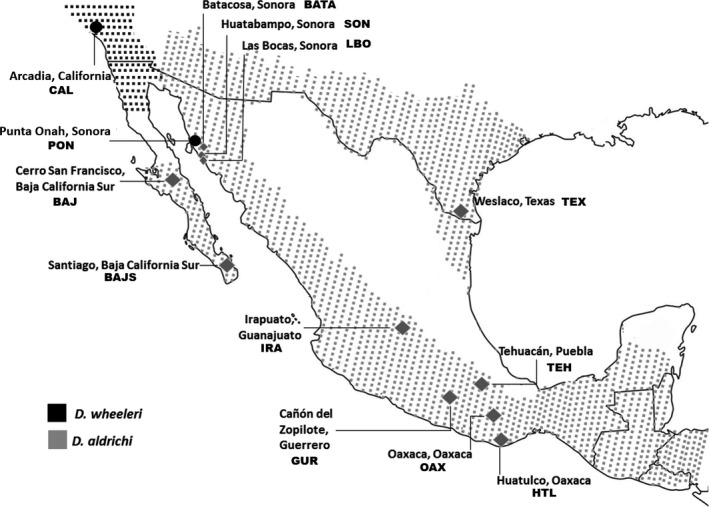
Recorded distributions of *Drosophila aldrichi* and *Drosophila wheeleri* in North America and *D. aldrichi* population localities used in experiments

Another cactophilic *Drosophila*, long assumed to be one species, is *D. aldrichi*, the sister species of *D. wheeleri* (Patterson & Alexander, 1952). While *D. wheeleri* is restricted in its distribution to coastal Southern California and the northern part of the Baja California peninsula, *D. aldrichi* is widespread in Mexico (Ruiz & Heed, 1988) and also has been reported from Central and South America. *Drosophila aldrichi* do not overlap in nature with *D. wheeleri* (Figure 1). Decaying pads of *Opuntia* cactus species provide the hosts for *D. aldrichi* (Ruiz & Heed, 1988), although anecdotal reports exist of associations with columnar cacti as well. No observable phenotypic differences have been reported among *D. aldrichi* from different parts of its range, even in collections from as far apart as Mexico and Central America, and thus it has been assumed to be one species.

Several lines of evidence, however, suggest that in fact, *D. aldrichi* is more than one species. Some of these indications are based upon crosses between different strains of *D. aldrichi*. For example, Richardson (1982) mentioned that crosses between strains of *D. aldrichi* from Texas and from Sonora yield sterile male offspring, although no data were provided. Wasserman (1992), in his extensive review of the repleta group, also reported that different strains of *D. aldrichi*, including those collected at the same locality, could not interbreed. Unfortunately, no data were provided in that report either. Subsequently, Krebs and Barker (1994) crossed *D. aldrichi* from Sinaloa with a strain from Australia, where they had been inadvertently introduced in the 1930s along with the *Opuntia*, their host plant. The specific North American origin of the Australian population was not known, but hybrid male sterility was observed in both directions. The information from Richardson (1982), Wasserman (1992) and from Krebs and Barker (1994) did not include any quantitative assessment of hybrid sterility or measures of sperm motility in the F1 males. Because the crosses among *D. aldrichi* strains produced offspring, sexual isolation was presumed to be absent. But here, again, no quantitative data were reported regarding the presence of premating incompatibilities. The fact that sterility was observed in reciprocal crosses, however, and not in only one direction, suggests the existence of a significant level of divergence between the tested *D. aldrichi* strains. In the case of the crosses between Australian and Mexican *D. aldrichi,* 50 years of separation in Australia is insufficient time for this level of isolation to evolve, leading Krebs and Barker to suggest that in North America, *D. aldrichi* already existed as multiple species. All *D. aldrichi* populations tested in reciprocal crosses with *D. wheeleri* produce sterile sons (Patterson & Alexander, 1952).

Preliminary molecular data also support the idea of more than one *D. aldrichi* lineage. Analyses based upon only 372 mt COI and 520 *nad2* indicate two separate lineages of *D. aldrichi* that Oliveira, Leonidas, Etges, O'Grady, and Desalle (2008) refer to as Eastern and Western clades. Curiously, in a number of cases, flies from the same geographic area fall into different clades suggesting that cryptic species may exist sympatrically and that “Eastern” and “Western” are not the most appropriate terms to describe the different lineages. More importantly, however, their data suggest that the two *D. aldrichi* lineages are paraphyletic with their sister species, *D. wheeleri*. Clouding the relationships among *D. aldrichi* strains and between D. *wheeleri* and *D. aldrichi,* is a study in which Beckenbach, Heed, and Etges (2008) reported up to 1% sequence variation between certain *D. aldrichi* strains in different mitochondrial genes, but detected no paraphyly with respect to *D. wheeleri*. These somewhat disparate results may reflect the fact that the two studies utilized different genes and they also differed in the number of informative sites in their sequences. Thus the true evolutionary relationships of *D*. *aldrichi* lineages and their relationship with *D. wheeleri* remain unresolved. And finally, also unresolved are the nature and degree of reproductive isolation among *D. aldrichi* populations, and how isolation corresponds to genetic lineage or degree of divergence.

While the above studies strongly point to the existence of more than one species of *D. aldrichi*, we lack a comprehensive picture of the evolutionary relationships among *D. aldrichi* and *D. wheeleri*, as well as among the *D. aldrichi* populations that show reproductive isolation. By knowing the phylogenetic relationships among *D. aldrichi* populations, we can characterize the types of isolating mechanisms present among them. The first step is to verify whether indeed *D. aldrichi* is paraphyletic with respect to *D. wheeleri* and to identify which *D. aldrichi* populations belong to which lineage. The second step is to test the prediction that divergent *D. aldrichi* lineages will show reproductive isolation, while those within the same lineage will not. In order to elucidate the relationship between *D. aldrichi* and *D. wheeleri*, we generated genome‐wide single nucleotide polymorphism (SNP) data using double‐digest restriction associated DNA sequencing (ddRADseq), as well as a mitochondrial gene tree of *D. aldrichi* from North America. We found that indeed, there are multiple lineages of *D. aldrichi* and that they are paraphyletic with respect to *D. wheeleri*. We then tested for behavioral and postzygotic isolation among strains of *D. aldrichi* from different lineages finding that, in general, the observed reproductive isolation among *D. aldrichi* occurs between and not within the lineages.

## 2 Materials and methods

### 2.1 Strains of *D. aldrichi*

The strains of *D. aldrichi* from Texas and various parts of Mexico used in our molecular and behavioral studies are shown in Table S1. Of the two strains of *D. wheeleri*, one was from the UCSD Stock Center, while the other was an unusual and isolated discovery of a *D. wheeleri* from Punta Onah in northern Sonora where it had likely blown across from the northern part of the Baja California peninsula which is the normal range of the species (Etges & Sloan, 2008). Living cultures of every *D. aldrichi* strain were no longer available at the time we tested for reproductive isolation, so we could only use a subset of the strains. We were able, however, to conduct crosses among living strains that were from different lineages in order to test the prediction that evolutionarily divergent flies will show reproductive isolation (Figure 1). Cultures were maintained in 8 dram glass vials containing medium prepared from instant mashed potato (Verde Valle) and juice from the ripe fruit of a prickly pear (*Opuntia)* cactus. Virgin males and females were separated upon emergence and stored in vials containing banana medium sprinkled with live yeast until used in reproductive isolation studies.

### 2.2 Phylogenetic studies

Total genomic DNA was isolated from individual adult flies using the procedure described in the DNeasy^®^ Blood and Tissue Kit (Qiagen). Whole flies were used for DNA extraction. Two mitochondrial genes were used for polymerase chain reaction (PCR) and sequencing: COI (cytochrome oxidase subunit 1) and COII (cytochrome oxidase subunit 2) (Table S2). PCR amplifications for COI and COII were performed using the following conditions: 95°C for 5 min, followed by 35 cycles of 95°C for 30 s, 55°C for 45 s, 72°C for 1 min and 72°C for 7 min. Purified PCR products (QIAquick^®^ PCR Purification Kit, Qiagen) were sequenced (Sanger sequencing, Servicios Genómicos LANGEBIO) and obtained sequences were aligned and corrected with Geneious^®^ software (Kearse et al., 2012). Sequences are deposited in GenBank with accession numbers KY700737‐KY700758.

Selection of best‐fit partitioning schemes and models of molecular evolution were performed using PartitionFinder software (Lanfear, Calcott, Ho, & Guindon, 2012), and concatenated gene analysis of COI and COII (1,159 bp) was performed in a Bayesian inference of phylogeny (MB) framework using MrBayes (Huelsenbeck & Ronquist, 2001). Both COI and COII were split by codon in order to select the appropriate substitution model. Three partitions were selected, one for each position of the codon in both genes. Substitution models for each partition were as follows: (1) First codon position: General Time Reversible (GTR), (2) Second codon position: Felsenstein (F81) and (3) Third codon position: Hasegawa‐Kishino‐Yano (HKY85+G). A haplotype network was built using statistical parsimony implemented in TCS (Clement, Posada, & Crandall, 2000) using PopART version 1.7 (http://popart.otago.ac.nz) in order to analyze the relationships between haplotypes in *D. aldrichi* populations. Uncorrected pairwise genetic distances of the combined COI and COII were calculated using PAUP* version 4.0 (Swofford 2002).

Samples used for RADseq libraries were obtained from the *Drosophila* Species Stock Center (Table S1). Library preparation for ddRADseq largely followed the protocol outlined by Peterson, Weber, Kay, Fisher, and Hoekstra (2012), and all quantification steps were done using a Qubit Fluorometer (Thermo Fisher Scientific). We extracted genomic DNA using Qiagen DNeasy Blood & Tissue kits (Qiagen, Inc.) using three female flies from each stock to obtain the necessary quantity of DNA for library preparation. We normalized all starting DNA concentrations so that each digest contained 500 ng of DNA. We used the enzymes *Sbf*1 and *Msp*1 (New England BioLabs) to digest the genomic DNA for 2 hr at 37°C. After digestion we purified the samples using Agencourt AMPure beads followed by a ligation step to attach Illumina adaptors to the digested DNA fragments prior to pooling. We size selected 415–515 bp DNA fragments of the pooled samples using a Pippin Prep fractionator (Sage Science) and ran a limited cycle PCR using Illumina indexing primers with a PhusionTM Polymerase kit (New England BioLabs). We assessed the final concentration and fragment size distribution of the pooled samples using a 2100 BioAnalyzer (Agilent Technologies). Samples were sent to the QB3 Vincent J. Coates Genomics Sequencing Laboratory at UC Berkeley, and an additional qPCR step was performed before pools were combined in equimolar concentrations and multiplexed (96 samples per lane) on an Illumina HiSeq 2500 (50‐bp, single end reads).

Illumina reads were demultiplexed, filtered, and analyzed using the pyRAD v3.0.5 software pipeline (Eaton 2014). The first round of filtering converted all base pairs with a PHRED score <20 to “N,” and any read with more than 6 “N”s was discarded. The mean depths of coverage ranged from 46–142. Within‐sample clustering was done in pyRAD using VSEARCH v.1.1.1 (www.github.com/torognes/vsearch) with a minimum clustering threshold of 87%, followed by among sample clustering of homologous loci using MUSCLE v3.8.31 (Edgar 2004) using the same clustering threshold. Consensus sequences within samples were constructed using the mean error and heterozygosity rates estimated from the data, and a minimum depth of coverage set to six. pyRAD then generated consensus sequences among samples, and we allowed missing data for 10% of the taxa. To correct for the use of multiple individuals per DNA extraction, we set the ploidy level to four. The nexus output file was run in MrBayes for 10 million generations using a GTR + I + G model. The RADseq data set included 3,461 SNPs.

### 2.3 Reproductive isolation

Postmating reproductive isolation was measured by performing reciprocal crosses among a subset of the *D. aldrichi* strains from the localities shown in Figure 1. Living cultures from all the localities were not available for experiments. Ten sexually mature virgin females and 10 virgin males were placed in vials with culture medium. Flies were transferred after 3 days to vials with fresh culture medium, and 3 days later the males were removed. All offspring, until the last emerging adult from a vial, were counted and sexed, using a Chi‐square test to examine gender bias in the offspring. In the majority of cross types, two replicates were performed. Even in those cases where we had only one replicate, several hundred progeny were produced. Between 28 and 70 sexually mature males were dissected, depending upon the cross, and the presence of motile sperm was scored. The presence of even one motile sperm was scored as “motile.”

Premating isolation was measured among the same strains using multiple choice tests. Ten pairs of sexually mature virgin flies, five from each of two strains, were placed in a clear Plexiglas Elens‐Wattiaux mating chamber (Elens & Wattiaux, 1964) and observed for 1 hr. Twenty‐four hours prior to the experiment, flies were lightly colored with micro‐fluorescent dust (U. S. Radium Corporation). A minimum of six replicates was conducted for each set of two strains, and the colors were alternated between replicates.

### 2.4 Statistical analysis

Chi‐square tests were conducted to analyze departures from random mating in the multiple choice tests. The joint isolation index (*I*) of Merrell (1950), female and male isolation indices (*I*_1_, *I*_2_) were calculated from each strain for the multiple choice tests according to following:

$$I=[\left(n_{11}+n_{22}\right)-\left(n_{12}-n_{21}\right)]/n$$

$$I_{1}=\left(n_{11}-n_{12}\right)/\left(n_{11}+n_{12}\right)$$

$$I_{2}=\left(n_{22}-n_{21}\right)/\left(n_{22}+n_{21}\right)$$

where *n*_11_ is the number of homospecific matings (females from Strain 1 and males from Strain 1), *n*_12_ is the number of heterospecific matings (females from Strain 1 and males from Strain 2 and vice versa), and *n* is the total number of matings. Standard errors (SE) of these indices were calculated by:

(1) $$\sqrt{\mathrm{SE}=\sqrt{(1-I_{2})/n}}$$

(Malagolowkin‐Cohen, Simmons, & Levene, 1965).

## 3 Results

### 3.1 Evolutionary relationships

**Figure 2:**
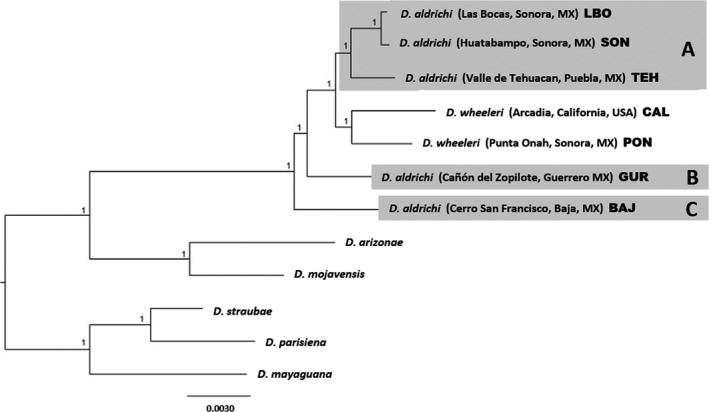
Phylogenetic analysis based on genome‐wide SNP data revealed three lineages, labeled A, B, and C

Earlier molecular studies disagreed about the relationship of *D. aldrichi* with its closest relative, *D. wheeleri*, with one suggesting that the closest relative of *D. aldrichi*,* D. wheeleri*, actually separates the former into two lineages. Although some nuclear data were utilized, these conclusions were based primarily on mtDNA due to lack of information in the nuclear markers (Oliveira et al., 2008). By using genome‐wide SNP markers, we were able to confirm that *D. aldrichi* is indeed paraphyletic with respect to *D. wheeleri* (Figure 2). The *D. aldrichi* lineages from the Mexican states of Sonora and Puebla were monophyletic (lineage “A”) and sister to *D. wheeleri*; however, the *D. aldrichi* lineages from Guerrero and Baja (lineages “B” and “C”) were sister to this clade resulting in paraphyly of *D. aldrichi*.

**Figure 3:**
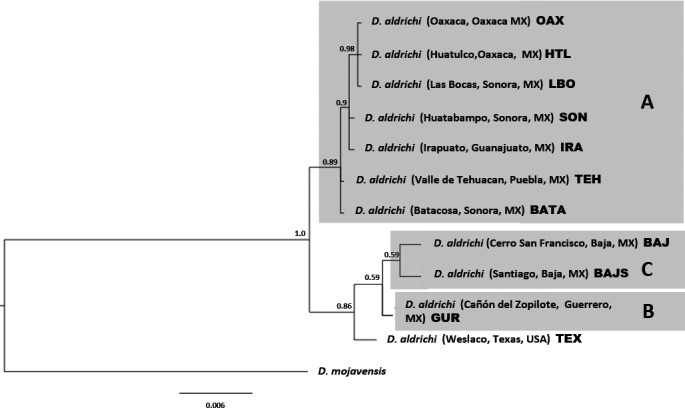
Phylogenetic analysis of *Drosophila aldrichi* based on concatenated mitochondrial COI and COII data (1,159 bp). Numbers above nodes are posterior probabilities recovered by the Bayesian analysis

A total of 1,159 nucleotide bp of concatenated mitochondrial COI and COII were analyzed for each *D. aldrichi* strain (Table S1). More strains were available for the mitochondrial analyses as it was performed prior to the SNP study. Phylogenetic relationships based on the Bayesian analyses are shown in Figure 3. These results also are consistent with the presence of two *D. aldrichi* lineages: one containing the peninsular strains (from Baja California), Texas, and Guerrero (monophyletic “B” and “C”); and one with the strains from the remaining mainland localities (lineage “A”). These two lineages have moderate support (0.89 and 0.86), but support within the Baja group is generally low with respect to relationships among strains.

**Figure 4:**
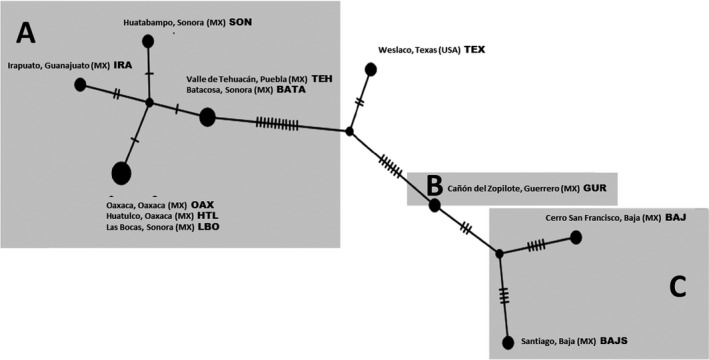
*Drosophila aldrichi* haplotype network based on concatenated mitochondrial COI and COII data (1,159 bp). Each tick mark represents a single nucleotide substitution

The haplotype network (Figure 4) reflects 15 bp differences between the Texas and Mexican mainland populations, with the exception of the Guerrero sequence, which is closer to the Baja sequences. Eight base pair changes separate Baja from Guerrero. Examining the % of sequence divergence (Table 1) reveals a similar pattern, with the greatest divergences between Baja and the southern Mexico strains and Texas and the southern Mexico strains, with the exception of the strain from Guerrero. The greatest sequence divergences, 1.64%‐1.9%, were between Guerrero and the other Mexican mainland strains.

**Table 1:** Average percent sequence divergence in 1,159 base pairs of COI and COII

	BAJ	BAJS	SON	LBO	BATA	TEX	IRA	TEH	GUR	OAX	HTL
BAJ		0.78	1.47	1.47	1.47	1.29	1.55	1.47	0.69	1.47	1.47
BAJS			1.55	1.55	1.38	1.38	1.64	1.38	0.60	1.55	1.55
SON				0.17	0.17	1.38	0.26	0.17	1.81	0.17	0.17
LBO					0.17	1.38	0.26	0.17	1.81	0.00	0.00
BATA						1.21	0.26	0.00	1.64	0.17	0.17
TEX							1.47	1.21	0.78	1.38	1.38
IRA								0.26	1.90	0.26	0.26
TEH									1.64	0.17	0.17
GUR										1.81	1.81
OAX											0.00
HTL											

### 3.2 Postzygotic reproductive isolation

Taken together these data predict that reproductive isolation, if present, should be observed between rather than within major lineages. Unfortunately living strains of *D. aldrichi* were not equally available for all lineages at the time of the experiments. We were able, however, to include those from Baja and Guerrero, along with four from the Mexican mainland.

Results of reciprocal homotypic crosses are presented in Table 2. Within populations, although female biased, sex ratios did not differ significantly from 1:1. No males were observed to lack motile sperm (Table 2a). In heterotypic crosses (Table 2b), when a F1 sex ratio was significantly different from 1:1, it was always a function of fewer sons. In one case the reduction in male progeny resulted from matings between Oaxaca mothers and Baja fathers. In three cases the reduction involved flies from Oaxaca and another case was in the cross between Tehuacan and Huatabampo.

**Table 2:** Sex ratios and number of F1 males with motile and nonmotile sperm in (a) homotypic and (b) heterotypic crosses of *Drosophila aldrichi*. Statistically significant differences in sex ratio, as measured by Chi‐square tests, are indicated by an *

(a)							
Cross		Reps	Progeny		χ^2^	Fertile males	
F	M		F	M		N w/motile sperm	%
BAJ	BAJ	2	201	173	2.1	70/70	100%
OAX	OAX	2	75	85	0.62	70/70	100%
GUR	GUR	2	218	165	6.08	70/70	100%
HTL	HTL	1	175	158	0.86	70/70	100%
TEH	TEH	2	228	209	0.82	70/70	100%
SON	SON	1	126	140	0.737	35/35	100%

(b)							
Cross		Reps	Progeny		χ^2^	Fertile F1 males	
F	M		F	M		N w/motile sperm	%
BAJ	GUR	2	488	465	0.55	41/70	59%
BAJ	OAX	2	137	157	1.36	36/70	51%
BAJ	HTL	2	390	377	0.22	51/70	73%
BAJ	TEH	2	159	143	0.84	17/70	24%
BAJ	SON	1	106	104	0.019	7/31	23%
GUR	BAJ	2	393	358	1.6	15/70	21%
GUR	OAX	2	261	234	1.47	70/70	100%
GUR	HTL	2	275	240	2.4	70/70	100%
GUR	TEH	2	306	291	0.37	70/70	100%
GUR	SON	1	193	164	2.35	26/26	100%
OAX	BAJ	2	284	223	7.33*	0/70	0%
OAX	GUR	2	402	431	1.01	70/70	100%
OAX	HTL	2	224	183	4.1*	70/70	100%
OAX	TEH	2	263	276	0.31	70/70	100%
OAX	SON	2	251	223	1.6	35/35	100%
TEH	BAJ	2	245	287	3.31	0/70	0%
TEH	GUR	2	230	235	0.054	70/70	100%
TEH	OAX	2	371	285	11.27*	70/10	100%
TEH	HTL	2	206	188	0.82	70/70	100%
TEH	SON	1	119	68	13.9*	28/28	100%
HTL	BAJ	2	265	283	0.6	2/70	3%
HTL	GUR	2	353	343	0.02	70/70	100%
HTL	OAX	2	321	271	4.22*	70/70	100%
HTL	TEH	2	180	174	0.10	70/70	100%
HTL	SON	1	131	116	0.91	35/35	100%
SON	BAJ	2	280	186	18.96*	0/38	0%
SON	GUR	2	178	167	0.35	35/35	100%
SON	OAX	2	68	54	1.6	35/35	100%
SON	TEH	2	194	173	1.2	35/35	100%
SON	HTL	1	113	113	‐	35/35	100%

All observations of F1 male sterility were observed in the crosses between flies from Baja peninsula and the other localities. When the fathers were from Baja, F1 males effectively had no motile sperm. When the mothers were from Baja, between 24% and 73% of F1 sons lacked motile sperm and those with motile sperm had limited numbers of them. The prediction that Guerrero also should show isolation from the other populations but not with Baja was not fulfilled. Baja females crossed with Guerrero produced only sterile males, just as in the crosses with other mainland males. At the same time, while the sons of Baja males crossed with the other lineage's female were all sterile, an intermediate number of sons from Guerrero females were fertile.

### 3.3 Sexual isolation

Results of multiple choice mating tests are presented in Table 3. Baja flies exhibited significant sexual isolation in pairings with all of the other localities, except with Guerrero. In most pairings there were significant excesses of females mating with males from their own populations. Males from the mainland were less successful with Baja females and more successful with their own. Within the mainland, however, things were different. Departures from random mating were not usually significant and isolation indices tended to be negative, indicating that nonrandom mating favored males from other mainland localities. In some cases, negative isolation indices were significant, as in crosses between Guerrero and Tehuacan or Huatulco, Tehuacan and Oaxaca, and Tehuacan and Huatulco.

**Table 3:** Multiple choice mating test results. χ^2^ tests were conducted to detect deviations from random mating (1:1:1:1). *I*(SE) is the joint isolation index. Significant sexual isolation exists when the index is twice as large as the SE (Malagolowkin‐Cohen et al., 1965; Zouros & D'Entremont, 1980). *I*_1_ indicate isolation due to females, *I*_2_ isolation due to males

Populations		*N*	A × A	A × B	B × A	B × B	χ^2^	*I* (SE)	*I*_1_ (SE)	*I*_2_ (SE)
A	B									
BAJ	GUR	81	26	20	13	22	4.38	0.19 (0.11)	0.26 (0.11)^a^	0.13 (0.11)
BAJ	OAX	81	30	15	9	27	14.55^a^	0.41 (0.10)^a^	0.33 (0.10)^a^	0.50 (0.10)^a^
BAJ	HTL	63	25	11	5	22	16.68^a^	0.49 (0.11)^a^	0.39 (0.12)^a^	0.63 (0.10)^a^
BAJ	TEH	77	22	15	13	27	6.48	0.27 (0.11)^a^	0.19 (0.11)	0.35 (0.11)^a^
BAJ	SON	73	23	13	10	27	10.67	0.37 (0.11)^a^	0.28 (0.11)^a^	0.46 (0.10)^a^
GUR	OAX	81	24	24	17	16	2.8	−0.01 (0.11)	0.00 (0.11)	−0.03 (0.11)
GUR	TEH	72	13	24	17	18	3.44	−0.14 (0.12)	−0.30 (0.11)^a^	0.03 (0.12)
GUR	HTL	63	10	21	19	13	5.0	−0.27 (0.12)^a^	−0.35 (0.12)^a^	−0.19 (0.12)
GUR	SON	67	15	18	19	15	0.76	−0.10 (0.12)	−0.09 (0.12)	−0.12 (0.12)
OAX	TEH	72	12	23	20	17	2.66	−0.19 (0.12)	−0.31 (0.11)^a^	−0.08 (0.12)
OAX	HTL	64	11	19	19	15	2.75	−0.19 (0.12)	−0.27 (0.12)^a^	−0.12 (0.12)
OAX	SON	66	9	17	19	12	4.18	−0.24 (0.12)^a^	−0.29 (0.12)^a^	−0.20 (0.12)
TEH	HTL	64	16	16	21	11	3.12	−0.16 (0.12)	0.00 (0.13)	−0.31 (0.12)^a^
TEH	SON	83	13	24	27	18	5.65	−0.24 (0.11)^a^	−0.30 (0.11)^a^	−0.20 (0.11)
HTL	SON	59	14	14	18	13	1.0	−0.08 (0.13)	0 (0.13)	−0.16 (0.13)

a indicates statistically significant isolation index.

## 4 Discussion

With respect to the evolutionary relationships among *D. aldrichi* populations, and between *D. aldrichi* and *D.wheeleri*, our SNP results reveal that *D. aldrichi* is indeed paraphyletic. While not only allowing us to demonstrate the paraphyletic relationship of *D. aldrichi* and *D. wheeleri*, the data also allow us to examine reproductive isolation among *D. aldrichi* in a more clearly defined phylogenetic framework. The haplotype network and genetic distances further refine the relationships because additional populations were available for mitochondrial gene sequencing. An obvious prediction is that greater reproductive isolation should be seen among versus within the paraphyletic *D. aldrichi* lineages and should reflect the degree of genetic divergence.

Living strains available for testing behavioral isolation and sperm motility fell into multiple paraphyletic lineages (A, B, and C in Figure 2). Previous studies demonstrated the complete isolation between *D. wheeleri* and *D. aldrichi* (Patterson & Alexander, 1952). But while earlier studies mentioned problems crossing various *D. aldrichi* populations from within North America (Richardson, 1982; Wasserman 1992), quantitative and qualitative data on the levels of reproductive isolation was not reported, and the evolutionary relationships among strains were unknown. We finally can examine the levels and nature of isolation observed among *D. aldrichi* in the context of their genetic divergence as determined by mitochondrial COI and COII, as well as genome‐wide SNPs. Indeed, as predicted, the greatest isolation observed was between flies from Baja and the majority of the mainland strains (lineage A). Baja males and mainland females always produced sterile sons. The exception was Guerrero, which, with respect to the mtDNA analysis, is in the same lineage as the Baja populations albeit with low support. The SNP data set, however, suggests a greater separation between the Guerrero and Baja populations, so the results of the reproductive isolation experiments are not surprising.

In crosses with Baja females and Guerrero males, there was reduction in sperm motility, but it was not as complete as in the reciprocal cross. When Guerrero females were crossed with the other strains, it is only with Baja males where the sons exhibit some motile sperm motility.

The same sort of pattern is observed in the behavioral isolation data: Baja exhibits lower isolation from Guerrero than from the other Mexican mainland. Isolation indices between Guerrero and the other mainland strains are largely negative.

During the speciation process, the first incompatibilities to arise are normally asymmetrical and influence male fertility and or viability, and typically appear in the males of one cross before they are observed in the reciprocal cross Coyne & Orr, 2004). We have several reasons to believe that the isolation between some lineages of *D. aldrichi* is not recent. One is the clear support for paraphyly and levels of genetic divergence. The average sequence divergence for the concatenated COI and COII regions between Baja and the all of the Mexican mainland strains is about 1.5%, with the exception of Guerrero. Another is the fact that in some reciprocal crosses a reduction in F1 sperm motility is seen in both directions, rather than one, consistent with a longer separation between the two lineages.

Our data strongly support the existence of at least two *D. aldrichi* species, one from mainland Mexico and one from the Baja peninsula. Baja males produce sterile sons when mated to females from the other localities, and there is a clear reduction in the number of males with motile sperm in sons of the reciprocal cross. Thus sterility appears to be bidirectional between Baja and other allopatric localities. Premating isolation, on the other hand, is significant in some cases but incomplete and thus, at least in the laboratory, represents a weaker and possibly later isolating mechanism than hybrid male sterility, at least in allopatry. Our study did not test for the existence of postmating–prezygotic isolating mechanisms, but this is of interest for future studies. The fact that hybrid females appear to be fertile suggests that backcrosses could be made among lineages to further elucidate the underlying genetics of the observed isolating mechanisms, especially hybrid male sterility.

Determining the degree of sequence typically divergence observed before reproductive isolation is detected is difficult to ascertain. For example, Cognato (2006) surveyed genetic divergence between a large number of insect sibling species and while the majority of closely related species pairs had sequence divergences between one and two percent, similar to what we found in *D. aldrichi*, many species in his survey had far greater divergences. One problem with comparisons to other *Drosophila* species, pointed out in our introduction, is that while many studies exist, the great majority employed older methodology such as allozyme frequencies or restriction enzyme analyses, impairing comparisons to our molecular sequence data. Comparable molecular sequence data do exist, however, for several other *Drosophila* species pairs. For example, the sibling pair *D. simulans* and *D. mauritiana*, which produce sterile F1 sons but fertile females (Coyne, 1984), have a sequence divergence of approximately 1% (Kingan, Geneva, Vedanayagam, & Garrigan, 2015). *Drosophila mojavensis* and its sister species *D. arizonae,* with estimated sequence divergence of between 1–2% (Reed, Nyboer, & Markow, 2007), produce sterile sons in only one direction of the cross, and they are defined as good species. If unidirectional hybrid male sterility defines species in the *D. simulans–D. mauritiana* (Coyne, 1984) and *D. mojavensis–D. arizonae* species pairs (Ruiz, Heed, & Wasserman, 1990), it would be appropriate to split the *D. aldrichi* from Baja and those from Sonora, Oaxaca, and Puebla into two separate species. The potential for historical hybridization between the Guerrero and Baja populations complicates this finding and necessitates additional studies before these species can be formally described and the placement of Guerrero determined. In addition, without living strains from Texas and Tamaulipas, we cannot eliminate the possible existence of more than two *D. aldrichi* species, especially given the climatic and geographic distances among these localities.
