# Supplementary material for: Early events in speciation: Cryptic species of Drosophila aldrichi
Source: Ecol Evol. 2017 May 3;7(12):4220–8. doi: 10.1002/ece3.2843 (PMC5478054; doi:10.1002/ece3.2843)
Supplement: Supplementary file 1 [file ECE3-7-4220-s001.docx]

Supplementary Table 1. Collection localities of *D. aldrichi* and *D. wheeleri* used in the present study. Strains in bold were used in the behavioral studies of reproductive isolation. In reference column, DSSC = UC San Diego Drosophila Species Stock Center. In molecular data column, COI and COII = mitochondrial data; GW = genome wide SNP data.

| **Species** | **Locality** | **Strain number** | **Reference** | **Molecular Data** |
| --- | --- | --- | --- | --- |
| ***D. aldrichi*** | **Cerro San Francisco, Baja California Sur** | **15081-1251.17** | **DSSC** | **COI, COII, GW** |
|  | **Oaxaca, Oaxaca** | **15081-1251.13** | **DSSC** | **COI, COII** |
|  | **Cañón del Zopilote, Guerrero** | **15081-1251.12** | **DSSC** | **COI, COII, GW** |
|  | **Valle de Tehuacán, Puebla** | **15081-1251.21** | **DSSC** | **COI, COII, GW** |
|  | **Huatulco, Oaxaca** | **15081-1251.22** | **DSSC** | **COI, COII** |
|  | **Huatabampo, Sonora** | **HTB-0515** | **Present study collection** | **GW** |
|  | Las Bocas, Sonora | 15081-1251.23 | DSSC | COI, COII, GW |
|  | Irapuato, Guanajuato | IRA-1214 | Present study collection |  |
|  | Weslaco, Texas | 15081-1251.01 | DSSC | COI, COII |
|  | **Batacosa, Sonora** | **BATA** | **Present study collection** | **COI, COII** |
|  | Santiago, Baja California | 15081-1251.10 | DSSC | COI, COII |
| *D. wheeleri* | Arcadia, CA, USA | 15081-1501.01 | DSSC | GW |
|  | Punta Onah, Sonora | NA | Etges Laboratory | GW |

Supplementary Table 2: Genes and primers used for phylogenetic analysis.

| Gene | Primer | Sequence (5´- 3’) | Reference |
| --- | --- | --- | --- |
| COI | LCO1490-F | GGTCAACAAATCATAAAGATATTGG | Folmer *et al*. 1994 |
|  | HCO2198-R | TAAACTTCAGGGTGACCAAAAAAT |  |
| COII | TL2-J-3037-F | ATGGCAGATTAGTGCAATGG | Simon *et al*. 1994 |
|  | TK-N-3785-R | GTTTAAGAGACCAGTACTTG |  |
