# Supplementary material for: Early events in speciation: Cryptic species of Drosophila aldrichi
Source: Ecol Evol. 2017 May 3;7(12):4220–8. doi: 10.1002/ece3.2843 (PMC5478054; doi:10.1002/ece3.2843)
Supplement: Supplementary file 2 [file ECE3-7-4220-s002.docx]

| Gene | Primer | Sequence (5´- 3’) | Reference |
| --- | --- | --- | --- |
| COI | LCO1490-F | GGTCAACAAATCATAAAGATATTGG | Folmer *et al*. 1994 |
|  | HCO2198-R | TAAACTTCAGGGTGACCAAAAAAT |  |
| COII | TL2-J-3037-F | ATGGCAGATTAGTGCAATGG | Simon *et al*. 1994 |
|  | TK-N-3785-R | GTTTAAGAGACCAGTACTTG |  |
